# Myocardial contractile function in survived neonatal piglets after cardiopulmonary bypass

**DOI:** 10.1186/1749-8090-5-98

**Published:** 2010-11-02

**Authors:** Theodor Tirilomis, Oliver J Liakopoulos, K Oguz Coskun, Marc Bensch, Aron-Frederik Popov, Jan D Schmitto, Friedrich A Schoendube

**Affiliations:** 1 Department for Thoracic, Cardiac, and Vascular Surgery, Goettingen University, Goettingen, Germany

## Abstract

**Background:**

Hemodynamic function may be depressed in the early postoperative stages after cardiac surgery. The aim of this study was the analysis of the myocardial contractility in neonates after cardiopulmonary bypass (CPB) and mild hypothermia.

**Methods:**

Three indices of left ventricular myocardial contractile function (dP/dt, (dP/dt)/P, and wall thickening) were studied up to 6 hours after CPB in neonatal piglets (CPB group; n = 4). The contractility data were analysed and then compared to the data of newborn piglets who also underwent median thoracotomy and instrumentation for the same time intervals but without CPB (non-CPB group; n = 3).

**Results:**

Left ventricular dP/dt_max _and (dP/dt_max_)/P remained stable in CPB group, while dP/dt_max _decreased in non-CPB group 5 hours postoperatively (1761 ± 205 mmHg/s at baseline vs. 1170 ± 205 mmHg/s after 5 h; p < 0.05). However, with regard to dP/dt_max _and (dP/dt_max_)/P there were no statistically significant differences between the two groups. Comparably, although myocardial thickening decreased in the non-CPB group the differences between the two groups were not statistically significant.

**Conclusions:**

The myocardial contractile function in survived neonatal piglets remained stable 6 hours after cardiopulmonary bypass and mild hypothermia probably due to regional hypercontractility.

## Introduction

The postoperative course after cardiac surgery in infants and children is in most cases uneventful. However, in some cases hemodynamic deterioration was observed early after surgery. The first characteristic change is regarding systemic blood pressure. The cause may be hypovolemia or reduced cardiac output. In clinical studies a significant reduction of cardiac index and stroke work index started at least two hours after cardiopulmonary bypass [1]. Management of hypovolemia requires infusions to maintain fluid balance. A fall in cardiac index results in inotropic support. Nevertheless, a hemodynamic unstable situation may result in combined treatment with blood, colloid, and crystalloid infusions and use of catecholamines with the goal to prevent further hemodynamic deterioration and to restore adequate organ perfusion.

Extracorporeal perfusion, hypothermia, myocardial ischemia, and reperfusion are some of the factors identified to be responsible for postoperative hemodynamic depression [2]. Very often the terms *hemodynamics *and *hemodynamic instability *are incorrect used equal to the terms *contractility *and *contractile depression*. Keeping this condition in mind, is the following question very important: is the cardiopulmonary bypass with mild hypothermia responsible for possible postoperative impairment of myocardial contractility in neonates? The aim of present study was the analysis of indices regarding myocardial contractility of the left ventricle.

## Materials and methods

The experimental protocol was approved by the Animal Care and Use Committees of the University of Göttingen and of the Government of the District of Braunschweig, Germany. All animals were handled according to the Federal Laws and to the guidelines of the American Physiological Society. Experimental preparation and protocol were performed under sterile conditions. Newborn piglets (younger than seven days of age) were examined. The mean body weight of the piglets was 2.9 ± 0.4 kg.

Anaesthesia was induced with azaperon (4 mg/kg; i.m.), ketamine (10 mg/kg; i.m.), and maintained with ketamin (6 mg/kg/h; i.v.), pentobarbital (5-10 mg/kg/h; i.v.), and inhaled isoflurane. Mechanical ventilation was performed through tracheostomy. After median sternotomy, exposure of the heart, and systemic application of heparin (300 U/kg), first a Millar pressure transducer-tip catheter was placed into the left ventricle (SPC-350, Millar Instruments Inc., Houston, TX, USA), and then a sonomicrometric piezoelectric crystal was implanted in the anterolateral left ventricular wall (Hugo-Sachs Elektronik-Harvard Apparatus, March-Hugstetten, Germany).

**Figure 1:**
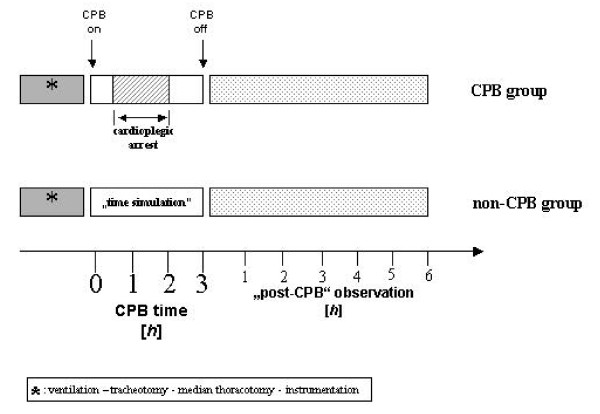
**Schematic presentation of time intervals in both groups**.

In the first group (CPB group) piglets were placed on CPB. In the second group (non-CPB group) three newborn animals were studied for the same time interval without cardiopulmonary perfusion (Figure 1).

Extracorporeal circuit was composed of a roller pump (Stöckert, Munich, Germany), a blood reservoir with pediatric membrane oxygenator (Babysafe, Jostra, Hirrlingen, Germany), and an arterial line blood filter (Capiox AFO2, Terumo Corp., Tokyo, Japan). The priming volume (300 ml) consisted of fresh whole neonatal piglet blood (two sibling animals per study animal), NaCl 0.9%, and 1000 units heparin. Cardiopulmonary bypass was initiated with a flow rate of 2.5 l/min/m^2^. Activated clotting time was maintained at a value >400 seconds throughtout duration of CPB.

On CPB, animals were cooled to 32°C core temperature. After 30 minutes the ascending aorta was crossclamped and cold *Bretschneider's *crystalloid cardioplegic solution (Custodiol HTK, Köhler Chemie, Alsbach-Hähnlein, Germany) was infused into the aortic root (30 ml/kg). Following 90 minutes of cardioplegic arrest, the aortic crossclamp was released and piglets were rewarmed to 37°C. After a total duration of 180 minutes animals were separated from CPB, cannulae were removed, and anticoagulation was reversed by protamine administration.

Thereafter piglets were observed for up to another six hours and data were registered.

No inotropic support was given throughout the protocol. Postoperative volume treatment was restrictive; central venous pressure (CVP) and left atrial pressure (LAP) were kept at the baseline levels (mean CVP <5 mmHg and mean LAP <2 mmHg, respectively).

Animals with complete observation time of six hours after termination of CPB were euthanized with an overdose of pentobarbital.

Contractility data analysis and calculations for CPB group (n = 4) and non-CPB group (n = 3) were performed regarding the following contractility parameters:

1) Left ventricular dP/dt_max_

2) Left ventricular contractility index (dP/dt_max_)/P, and

3) Changes in regional left ventricular myocardial thickening.

Twenty subsequent values were calculated for each time point per piglet. Data were expressed as mean ± standard deviation and processed with Statistica 6.1 software (StatSoft (Europe) GmbH, Hamburg, Germany). The data were analyzed by ANOVA, followed by Fisher's LSD procedure for post hoc repeated measurements. Differences were considered statistical significant at *P *< 0.05.

## Results

In the non-CPB group the left ventricular dP/dt_max _decreased from 1761 at baseline to 1170 mmHg/s at the endpoint (*P *< 0.05) (Table 1). The dP/dt_max _remained stable in the CPB group during follow up of six hours after the end of CPB and was similar to the baseline values (Table 1).

**Table 1:** Values of left ventricular dP/dt_max _[mmHg/s] before and after CPB (up to 6 hours) or time equivalent in non-CPB group.

group	*pre CPB (baseline)*	*CPB end*	*1 h post CPB*	*2 h post CPB*	*3 h post CPB*	*4 h post CPB*	*5 h post CPB*	*6 h post CPB*
**CPB **(n = 4)	1495 ± 159	1679 ± 159	1838 ± 159	1708 ± 159	1609 ± 159	1412 ± 159	1730 ± 180	1400 ± 180

**non-CPB **(n = 3)	1761 ± 205	1566 ± 205	1544 ± 205	1519 ± 205	1455 ± 205	1340 ± 205	1170 ± 205 *	1151 ± 205 *

* p < 0.05 vs. baseline

The performance of contractility index (dP/dt_max_)/P was in both groups more stable (Table 2).

**Table 2:** Left ventricular contractility index ((dP/dt_max_)/P) [/s] before and after CPB (up to 6 hours) or time equivalent in non-CPB group.

group	*pre CPB (baseline)*	*end CPB*	*1 h post CPB*	*2 h post CPB*	*3 h post CPB*	*4 h post CPB*	*5 h post CPB*	*6 h post CPB*
**CPB **(n = 4)	60.5 ± 4.1	67.1 ± 7.6	65.2 ± 10.6	63.9 ± 11.6	63.3 ± 12.1	62.8 ± 12.0	65.7 ± 11.7	65.4 ± 12.5

**non-CPB **(n = 3)	65.7 ± 3.5	78.3 ± 7.5	74.3 ± 6.7	75.0 ± 8.3	74.4 ± 9.1	71.5 ± 10.6	66.4 ± 21.3	65.2 ± 23.1

Myocardial thickening decreased significantly in non-CPB controls after the 2^nd ^hour "post-bypass" while it remained constant in CPB group (Table 3).

**Table 3:** Changes in (left ventricular) myocardial thickening [mm/s] before and after CPB (or time equivalent in non-CPB group).

group	*pre CPB (baseline)*	*end CPB*	*1 h post CPB*	*2 h post CPB*	*3 h post CPB*	*4 h post CPB*	*5 h post CPB*	*6 h post CPB*
**CPB **(n = 4)	1.21 ± 0.08	1.00 ± 0.08	1.00 ± 0.08	1.13 ± 0.08	1.08 ± 0.08	0.99 ± 0.08	1.03 ± 0.09	1.05 ± 0.09

**non-CPB **(n = 3)	1.45 ± 0.10	1.30 ± 0.10	1.23 ± 0.10	0.98 ± 0.10 *	1.01 ± 0.10 *	1.04 ± 0.10 *	1.01 ± 0.10 *	0.99 ± 0.10 *

* p < 0.05 vs. baseline

**Figure 2:**
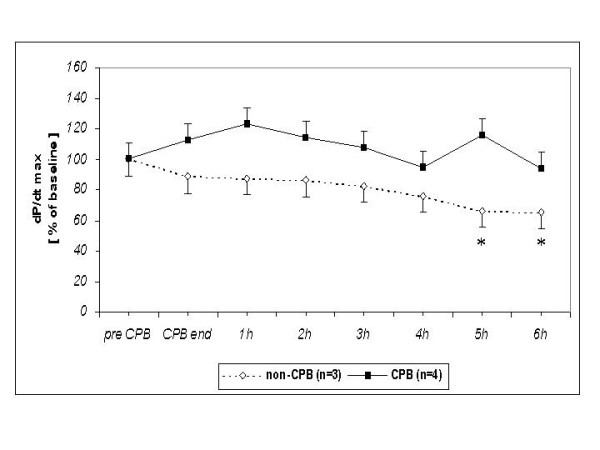
**Performance of the left ventricular dP/dt**_**max **_**in survived neonatal piglets in % of baseline value**. * *P *< 0.05 in comparison to the baseline value. No statistically significant differences between the two groups.

**Figure 3:**
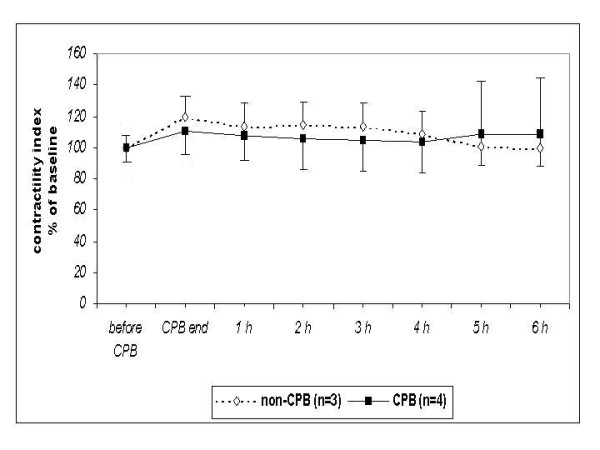
**Changes of the left ventricular contractility index (dP/dt**_**max**_**)/P in survived newborn piglets in % of baseline value**. No statistically significant differences between both groups.

**Figure 4:**
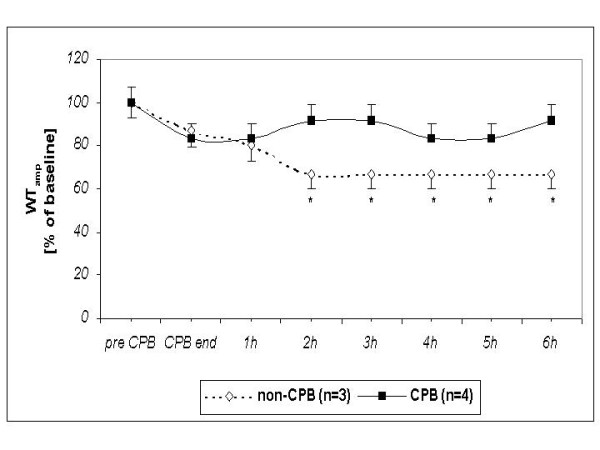
**Presentation of the changes of the left ventricular myocardial wall thickening (WT**_**amp**_**) in survived newborn piglets in % of baseline value**. * *P *< 0.05 in comparison to the baseline value. No statistically significant differences between the two groups.

The differences between the CPB and non-CPB group were not statistically significant regarding left ventricular dP/dt_max _(Figure 2), contractility index (dP/dt_max_)/P (Figure 3), and regional wall thickening (Figure 4).

## Discussion

The current study employed an *in vivo *neonatal piglet model in which clinical standard techniques used at our institution were applied. Many studies that examined myocardial contractility were performed on isolated hearts (modified Langendorff preparations) [3-6]. Extrapolation of results from these studies to the clinical situation should be viewed with caution. Therefore, present study provides more relevant information about myocardial contractility of the neonatal heart in a clinical setting. Furthermore, all piglets were within the age of the first week, before transition from the neonatal to the adult situation may result [7].

Additionally, the changes at birth consist of conversion from the fetal cardiovascular system to closure of low-resistance vascular pathways [8]. Functional closure of the ductus arteriosus occurs within 4 hours after birth [9]. At autopsy, we carefully examined the atrial septum and the ductus arteriosus, and they were never open.

The contractility parameter dP/dt_max _is a function of the contractile element power, the elasticity constant, and the ventricular dimensions [10]. Inotropic interventions (positive and negative) at constant end-diastolic volumes reflect changes in maximal contractile element power. In the present study there was no application of any positive inotropic drug avoiding pharmacological increase of myocardial contractility. The only drugs used were the anesthetics without differences in dosages between the two groups.

Increasing ventricular filling has two opposing effects on dP/dt_max_; (1) the volume increase tends to increase dP/dt_max_, according to Frank-Starling mechanism and (2) the greater volume tends to decrease it, in accordance to La Place effect. At physiological filling pressures, the first mechanism predominates [11]. In the current study volume treatment was restrictive; central venous and left atrial pressures were kept at the level before procedure; mean central venous pressure was less than 5 mmHg and mean left atrial pressure less than 2 mmHg.

However, application of dP/dt_max _may be limited, because of it load dependence. In this study, also the maximal values of the ratio of the first derivative of left ventricular pressure to instantaneous pressure (that is (dP/dt_max_)/P; so-called contractility index) have been considered. Peak values of (dP/dt_max_)/P were essentially independent of preload and afterload [12]. Nevertheless, extreme elevations of preload and afterload may decrease contractility index. Decrease of (dP/dt_max_)/P has been demonstrated for end-diastolic pressures >25 mmHg [13]. At aortic diastolic pressures of less than 120 mmHg, contractility index is independent of afterload [12]. In the present study preload and afterload remained within physiological range.

On a cellular level myocardial contractility depends on many factors such as sarcoplasmic reticulum calcium handling and myofilament calcium sensivity [14]. The sarcoplasmic reticulum seems to play a key role; the primary function of it is to accumulate and store calcium during diastole and release that calcium rapidly at the onset of systole, enabling the cardiomyocyte to develop rapid contraction [15]. Neonatal hearts reperfused after the development of peak ischemic contracture have shown negligible postischemic functional and metabolic recovery [16]. Our findings suggest that in a clinically relevant setting ischemic contracture and subsequent metabolic response could be avoided. The performance of wall thickening indicates in some degree of hypercontractiliy after CPB. This hypercontractility may be the result of the systemic inflammatory response on myocardial level.

The present study has two important limitations; (1) the inclusion of survived piglets only and (2) the duration of the post-bypass observation time of six hours, then decrease of myocardial contractility may result at least theoretically even later than six hours after CPB termination. Nevertheless, Burrows et al.[1] found deterioration of cardiac performance four hours after cardiopulmonary bypass for ventricular septal defect repair, Mustard's operation, and repair of Tetralogy of Fallot.

In general, the results of present study are surprising. From the theoretical point of view the response of neonatal myocardium to the effects of anaesthetic drugs may be modified after cardiopulmonary bypass resulting in this paradox of decreased myocardial contractility in the control group (non-CPB group). Additionally, the effect of the cardioplegic solution is not clear. The role of the applied cardioplegic *Bretschneider's *solution has to be elucidated in further studies comparing different types of myocardial protection.

## Conclusions

Applying an *in vivo *neonatal piglet model closely mimicking the clinical setting of cardiopulmonary bypass with mild hypothermia (and crystalloid cardioplegic myocardial protection) but without postoperative inotropic support, we found that the myocardial contractility of the neonatal heart remained in survived animals at the baseline values after cardiopulmonary bypass, probably due to some degree of regional hypercontractility.

## Competing interests

The authors declare that they have no competing interests.

## Authors' contributions

TT conceived the study, participated in design and coordination, participated in acquisition, analysis and interpretation of the data and drafted the manuscript. OJL participated in the design of the study and performed the statistical analysis. KOC participated in data analysis and helped to draft the manuscript. MB participated in the design of the study and helped in acquisition of the data. AFP participated in data analysis and helped to draft the manuscript. JDS participated in data analysis and helped to draft the manuscript. FAS participated in the design and coordination, and revised manuscript critically. All authors read and approved the final manuscript.
